# Urinary Extracellular Vesicles as a Readily Available Biomarker Source: A Simplified Stratification Method

**DOI:** 10.3390/ijms25158004

**Published:** 2024-07-23

**Authors:** Lidija Filipović, Milica Spasojević Savković, Radivoje Prodanović, Suzana Matijašević Joković, Sanja Stevanović, Ario de Marco, Maja Kosanović, Goran Brajušković, Milica Popović

**Affiliations:** 1 Innovative Centre of the Faculty of Chemistry, 11158 Belgrade, Serbia; filipoviclidija29@gmail.com (L.F.); smilica84@gmail.com (M.S.S.); 2 Faculty of Chemistry, University of Belgrade, 11158 Belgrade, Serbia; rprodano@chem.bg.ac.rs; 3 Faculty of Biology, University of Belgrade, 11158 Belgrade, Serbia; suzana.matijasevic@bio.bg.ac.rs (S.M.J.); brajuskovic@bio.bg.ac.rs (G.B.); 4 Center for Chemistry, Institute for Chemistry, Technology, and Metallurgy, National Institute of Republic of Serbia, 11000 Belgrade, Serbia; sanjas@ihtm.bg.ac.rs; 5 Laboratory for Environmental and Life Sciences, University of Nova Gorica, 5000 Nova Gorica, Slovenia; ario.demarco@ung.si; 6 Institute for the Application of Nuclear Energy, INEP, University of Belgrade, 11080 Belgrade, Serbia; maja9129@gmail.com

**Keywords:** extracellular vesicles, nanobodies, immunoaffinity chromatography, ultracentrifugation

## Abstract

Urine, a common source of biological markers in biomedical research and clinical diagnosis, has recently generated a new wave of interest. It has recently become a focus of study due to the presence of its content of extracellular vesicles (EVs). These uEVs have been found to reflect physiological and pathological conditions in kidney, urothelial, and prostate tissue and can illustrate further molecular processes, leading to a rapid expansion of research in this field In this work, we present the advantages of an immunoaffinity-based method for uEVs’ isolation with respect to the gold standard purification approach performed by differential ultracentrifugation [in terms of purity and antigen presence. The immunoaffinity method was made feasible by combining specific antibodies with a functionalized polymethacrylate polymer. Flow cytometry indicated a significant fluorescence shift, validating the presence of the markers (CD9, CD63, CD81) and confirming the effectiveness of the isolation method. Microscopy evaluations have shown that the morphology of the vesicles remained intact and corresponded to the expected shapes and dimensions of uEVs. The described protocol is inexpensive, fast, easy to process, has good reproducibility, and can be applied to further biological samples.

## 1. Introduction

Extracellular vesicles (EVs) represent a heterogeneous group of lipid bilayer-derived structures produced by nearly all cell types [1,2]. They can be found in various bodily fluids, including ascites, breast milk, urine, saliva, and blood [3]. EVs are crucial in both physiological and pathological processes of intercellular communication [4]. They carry different cargo, including proteins, receptors, nucleic acids, and lipids. Based on their cellular origin and dimension, extracellular vesicles can be divided into three main groups: apoptotic bodies (50–5000 nm), microvesicles (50–1000 nm), and exosomes (40–200 nm) [5,6].

The most reliable method for identifying and tracking various urological conditions is biopsy. However, this procedure is invasive, so alternative diagnostics based on liquid biopsy would be highly welcome to diagnose urinary diseases [7]. Liquid biopsies are sampling and analysis of patient biological fluids of different origins to obtain disease-related information [8]. Urine is a highly accessible and informative biofluid, and its fast sampling can be carried out independently by patients. Numerous metabolites, proteins, cells, and cellular components found in urine are used to evaluate individual pathological and physical conditions [9]. These components mainly originate from the urinary tract and may be filtered directly from the systemic circulation, such as in the case of miRNAs [10]. Outside of total urinary analysis, urinary-derived EVs (uEVs) are promising cellular candidates for reliable biomarker identification. Indeed, uEVs have been studied in several pathological states, such as polycystic kidney disease [11], glomerular disease [12], tubulopathies [13], AKI [14], and prostate cancer. Moreover, the analysis of uEVs has the potential to lead to the discovery of further biomarkers that are meaningful for diagnosing and monitoring various urogenital conditions.

In this context, the main challenge in studying uEVs is their purification and characterization methods. Conventional techniques for EVs isolation are typically based on their size and include SEC, ultrafiltration, and ultracentrifugationUltracentrifugation is the most common method for EV isolation and exploits differential centrifugation to separate EVs from other components. This method requires specialized equipment since a very high speed (100,000–110,000× *g*) is necessary. It is also time-consuming because the pellet must be dissolved and centrifuged again to increase the sample purity [15]. Furthermore, this method cannot distinguish between uEVs of the same size but different origins and often results in samples contaminated with non-vesicular material. Alternative methods for purifying uEVs have been proposed [8,16], among which immune-affinity is particularly appealing. In this case, uEVs are selectively captured by means of specific antibodies that recognize some molecular targets displayed on the uEV surface. Conventional antibodies bound to magnetic beads have been used successfully for the purification sub-groups of EVs. This strategy results in good separation of EV sub-populations, but the amount of vesicles can be insufficient for downstream analyses [17]. A mixture of several monoclonal antibodies has been proposed to maximize the possibility of recovering uEV variants. Nevertheless, this strategy expands the production costs. Single-domain antibodies (VHH) represent an excellent alternative to monoclonal antibodies because they are structurally stable, the cost of production is significantly lower, they are simple to label at specific residues, and, as recombinant molecules, their characteristics and yields remain stable over time. Large pre-immune libraries can be used for direct panning procedures on different antigens (soluble antigens, whole cells, or EVs) [18,19]. The VHHs obtained by direct panning on EVs enable the capture of the vesicles from various sources [20]. The preparative purification of EVs from large amounts of complex samples cannot be achieved using conventional chromatographic material such as Sepharose beads because some of the bound material and some contaminants tend to clog the column. One of the alternatives is producing a macroporous copolymer, which has a more rigid structure and can be functionalized with desired antibodies [21,22]. Copolymers based on glycidyl methacrylate are more suitable for different immobilization because they can be easily transformed into hydroxyl, carboxyl, keto, or amino groups to facilitate binding antibodies for the carrier [23]. Recently, we demonstrated that methacrylate polymer functionalized with nanobodies effectively captures and releases EVs [21]. Most of the work for optimization methods for EVs isolation has been performed using cell culture medium and serum because they are sources with abundant EVs. In this work, we tried to implement previously developed immune-affinity methods for the purification of EVs from urine.

## 2. Results

### 2.1. Optimization Immunoaffinity Approach for Isolation of Urinary EVs

We tested two different elution methods for the isolation of urinary EVs from polymethacrylate carriers, one based on continuative elution from the column and the second in a batch after a longer incubation time. The resulting EVs were evaluated by NTA and flow cytometry.

**Figure 1:**
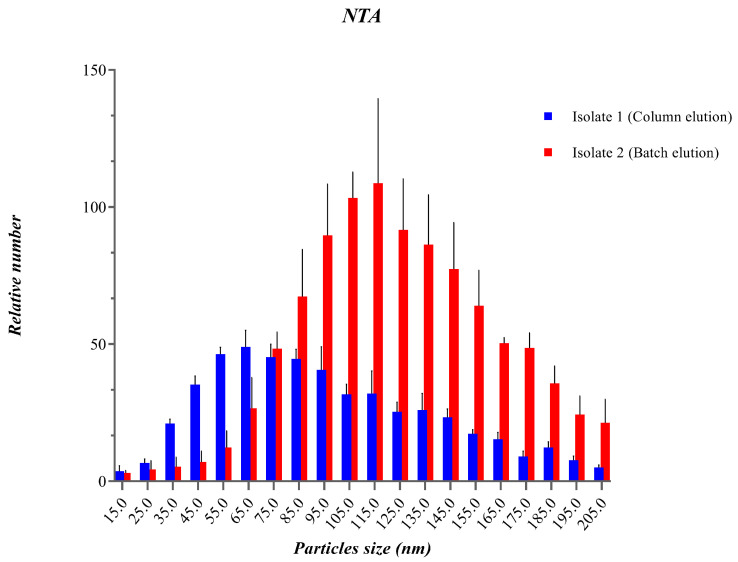
uEVs characterized using nanoparticle tracking analysis (NTA). The blue bars represent uEVs eluted progressively from the column, and the red bars correspond to uEVs recovered after batch incubation (45 min total). We plot the relative number and size distribution.

Analysis of the concentration and size distribution of uEVs by NTA showed that the elution method can affect the composition of isolated uEVs. Isolate 1 obtained using column elution (blue bars Figure 1) exhibited a particle size distribution centered between 35 nm and 125 nm, with a peak around 70 nm. Column elution resulted in a smaller average diameter of particles, 100.12 ± 15.9 nm. Isolate 2 obtained using batch elution (red bars Figure 1) exhibited a broader particle size distribution ranging from 35 nm to 215 nm, with a peak around 100 nm and a slightly larger average uEV diameter, 124.8 ± 2.47 nm. Column elution produced 2.49 × 10^9^ vesicles/mL, while batch elution provided 1.16 × 10^10^ vesicles/mL (Figure 1; Table 1).

**Figure 2:**
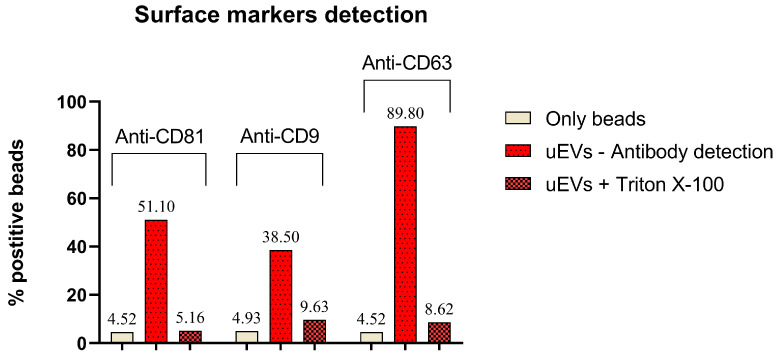
uEV surface markers detected in urine isolates. Isolated uEVs were immobilized on latex beads, which were analyzed for surface antigens: (Left Anti-CD63-AlexaFluor488, (Middle) Anti CD9-PE, and (Right) Anti-CD81 PE/Dazzle. Latex beads without uEVs were used as a negative control. The treatment with the detergent Triton X-100 reduced the signal, confirming that it was due to the presence of EVs.

Purified uEVs eluted in the batch were immobilized on latex beads, and flow cytometry was carried out to verify the presence of common uEV surface markers such as CD63, CD9, and CD81 (Figure 2; Supplementary Figure S1). The tested isolates showed a significant shift in fluorescence signal upon binding of specific antibodies. The percentages of fluorescent beads for anti-CD81, anti-CD9, and anti-CD63 were 51.10%, 38.50%, and 89.80%, respectively (Figure 2). Detergent controls were performed using TRITON X-100 in a final concentration of 0.5% (*w*/*v*). Upon incubation with Triton X-100, performed to remove the uEVs from the beads, the signal was significantly reduced to 5.16%, 9.6%, and 8.62% for the CD9, CD69, and CD81, respectively, namely values comparable to the background signals. Signal reduction upon detergent treatment confirmed that it was specifically due to uEVs (Figure 2; Supplementary Figure S1).

### 2.2. Comparison of Two Different Isolation Methods for Urine Extracellular Vesicles

After the isolation of uEVs with IM and CFG, we tested protein content with BCA assay and lipid with SPV assay (Table 2). In comparison to the IM method, which yielded proteins at a concentration of 0.279 mg/mL, the CFG method yielded 1.349 mg of protein per mL.

#### 2.2.1. Nanoparticle Tracking Analysis

**Figure 3:**
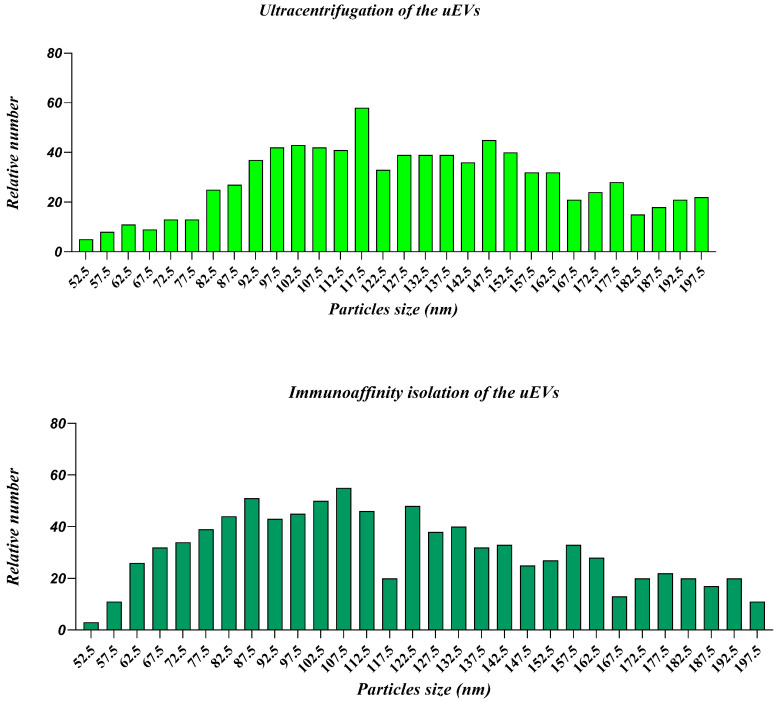
NTA of uEVs isolated by two different methods, ultracentrifugation (CFG) and the immunoaffinity approach (IM). Green bars (darken) represent uEVs by IM, and green (lighter) bars represent uEVs by CFG.

The relative number of vesicles (Figure 3 and Table 3) obtained using the two different isolation methods, immunoaffinity (IM) and ultracentrifugation (CFG), is in the same order of magnitude, namely 10^9^ particles/mL. The IM method yields a higher number of smaller particles, especially in the range between 52.5 nm and 112.5 nm, with the peak occurring between 97.5 nm and 107.5 nm. The number of particles gradually decreases as the particle size increases, yet a significant number of particles is still observed up to 197.5 nm. Ultracentrifugation also shows a high number of particles in the range from 87.5 nm to 137.5 nm, with a peak around 117.5 nm. However, the relative number of particles is generally lower than the IM method within the same size range. Compared to the IM method (4.20 × 10^9^ particles/mL), the CFG method yields a slightly higher amount of particles (8.00 × 10^9^ particles/mL), but apparently, they are more contaminated as the concentration of proteins is significantly higher.

#### 2.2.2. Flow Cytometry

**Figure 4:**
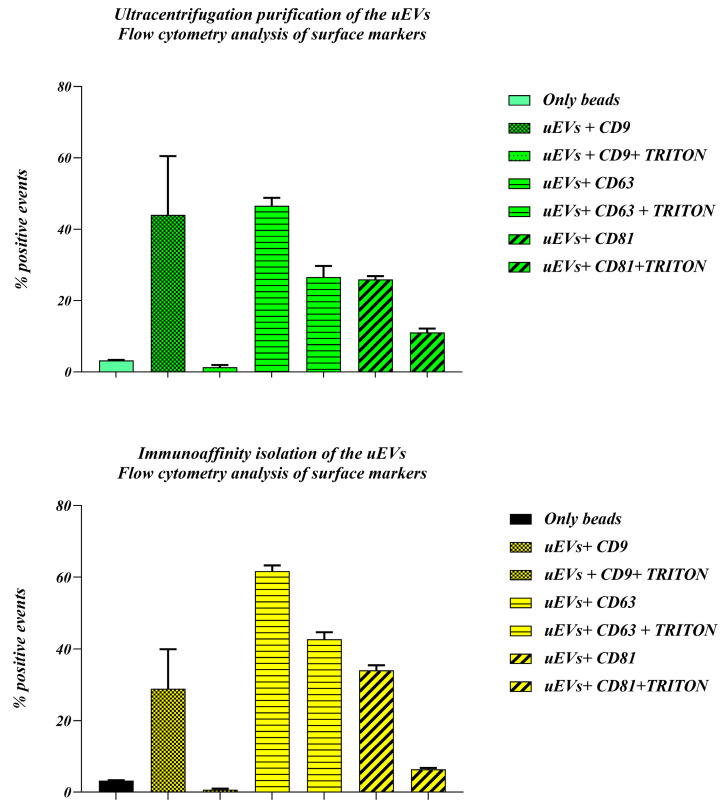
Comparison of the membrane biomarkers of uEVs isolated with two different methods. Three common surface markers were targeted with specific antibodies: Anti-CD63-AlexaFluor488, Anti-CD9-PE, and Anti-CD81 PE/Dazzle.

Urinary EVs obtained with two different methods (CFG and IM) were analyzed using flow cytometry for confirmation of the presence of the surface antigens (Figure 4). Ultracentrifugation shows significant positivity for CD9 (40%), CD63 (55%), and CD81 (35%), with Triton X-100 treatment reducing these values to around 5%, 45%, and 15%, respectively, confirming vesicular localization of these markers. Similarly, immunoaffinity isolation highlights CD63 as the most abundant marker (70%), followed by CD81 (45%) and CD9 (25%), with Triton X-100 treatment reducing positivity to 60%, 10%, and 5%, respectively. CD63 is the most prominent marker in both samples (Figure 4), indicating that it is a robust marker in the case of urinary vesicles. The slight variations in the percentages of positive events between the two purification methods could be attributed to differences in the isolation efficiency and purity of the uEV populations. Therefore, there is a difference in the positive events in the immunoaffinity approach of the pulled urine versus urine collected from one donor, which we represented in the first part of the results, probably due to different sample types.

### 2.3. Qualitative Analysis of Isolated EVs

#### 2.3.1. Scanning Electron Microscopy

**Figure 5:**
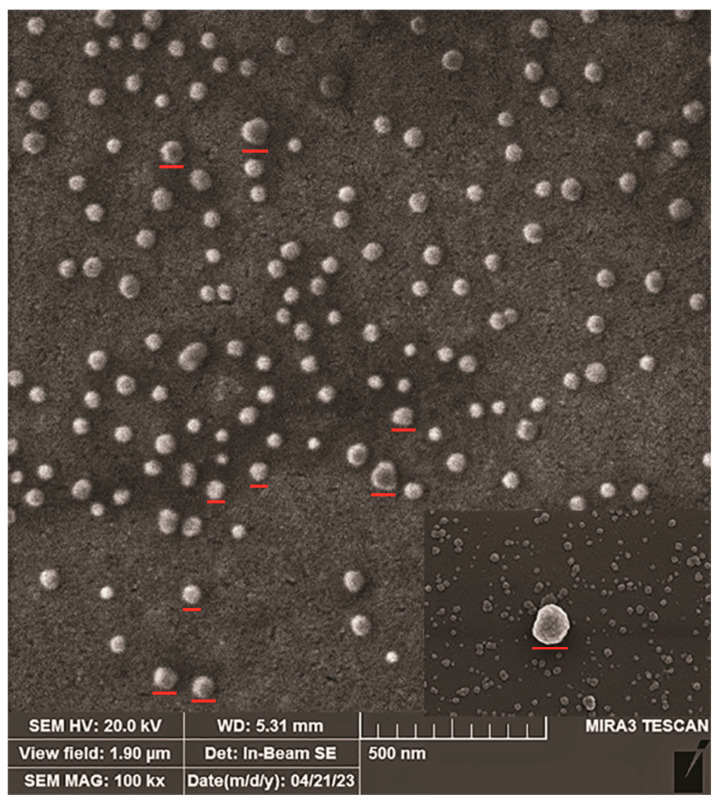
SEM analysis of immuno-purified uEVs after gold staining at a magnification of 100,000×. The figure shows that diameters are in the range of 30–150 nm. Red lines mark representative uEVs on the sample.

The morphology of the batch-isolated vesicles was verified by SEM. Several EVs can be seen throughout the field, appearing spherical in shape (Figure 5), with a size ranging from 20 to 115 nm. A magnified single EV is reported in the inset image in the lower right corner, highlighting its morphology. The EVs’ sizes and shapes throughout the field indicate that the isolation procedure did not significantly affect morphology nor lead to EV lysis.

#### 2.3.2. Atomic Force Microscopy

**Figure 6:**
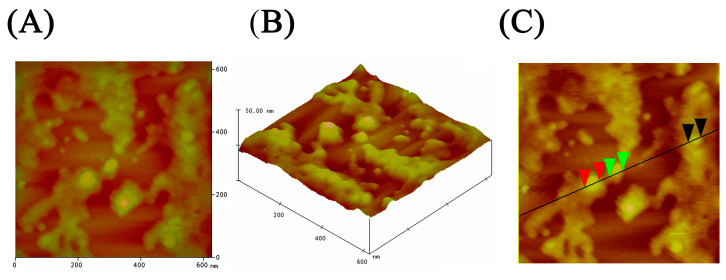
Characterization of uEVs with AFM. Figures (**A**,**B**) represent two-dimensional and three-dimensional analyses. Figure (**C**) represents a cross-sectional profile analysis.

AFM was used to further examine the surface morphology of isolated uEVs after their deposition on an atomically flat mica surface (Figure 6). Panels A and B show typical two-dimensional (2D) and three-dimensional (3D) pictures of isolated uEVs. The spherical or globular shape of uEVs can be seen clearly. The size of globules (spheres) was determined from an AFM image with cross-sectional profile analysis, as presented in panel C. The diameters of these globules range from 40 to 60 nm (red markers on the profile line denote globules of 51.37 nm in diameter, while green markers and black markers denote globules of 46.47 nm and 41.58 nm in diameter, respectively).

### 2.4. Testing the Reproducibility of the Immunoaffinity Approach

**Figure 7:**
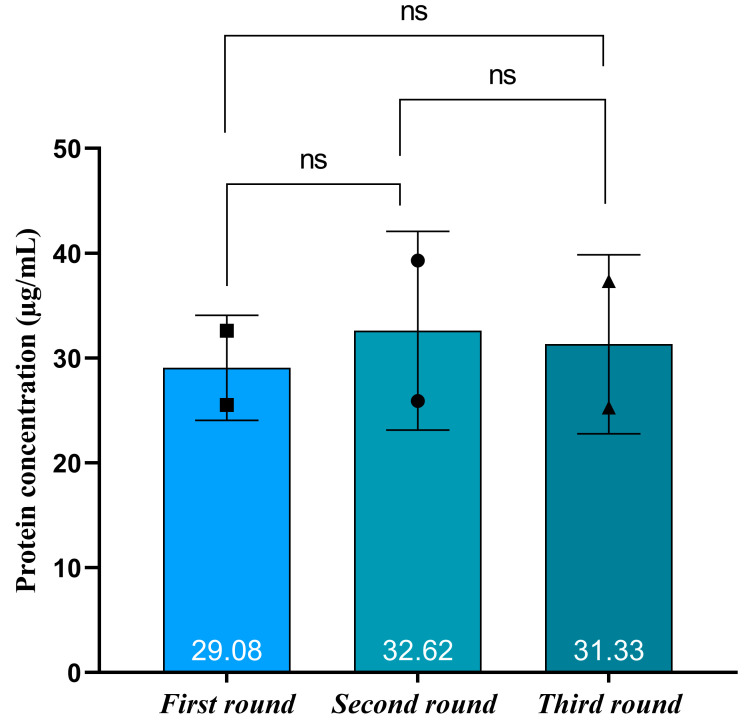
Testing the reproducibility of the immunoaffinity approach. The protein concentration in three independent isolates from the same urine pool is compared. Two squares/circles/triangles represent duplicate values of measured protein concentration. The number in the lower part of the bars represents the average value. The black lines represent standard error bars. The ns represent statistic comparation (no significant).

The methodology reproducibility was tested using the same urine pool and isolated three individual rounds of immunoaffinity chromatography (Figure 7), as we described in the part of material and methodology. The results demonstrate the method consistency since the isolate mean protein concentration remained constant (31.03 µg/mL), with no significant difference between the isolates (Figure 7).

## 3. Discussion

Finding biological fluids’ disease biomarkers that can be measured inexpensively for early disease diagnosis is an important goal in clinical practice and requires appropriate techniques for reducing the complexity of bodily fluid samples and identifying relatively low-abundance components that might have unique pathophysiological significance. Proteomics of blood serum or plasma has received the majority of the focus so far. Urine is a desirable substitute for blood plasma as a possible source of disease biomarkers because it can be collected noninvasively and in large quantities [24]. EVs are released by almost all cell types and are readily available in bodily fluids like blood, saliva, and urine, representing ideal biomarkers for liquid biopsy procedures that can substitute invasive methods in clinical practice [25]. The first time that urinary EVs were reported as carrier-important biomarkers was in 2009 [26] Rapid expansion in the field of EVs has made the development of methods for their isolation in significant quantities an important prerequisite [27,28]. The large majority of researchers use ultracentrifugation to separate EVs across all applications, and this method still represents the “gold standard” [29] The increasing interest in EVs has demanded the implementation of alternative methods to standard ultracentrifugation [30,31]. Immunoaffinity methods combine specific recognition of specific antigens on the surface of vesicles. The most frequently used targets are tetraspanins, which facilitate exosome isolation. The solid phase, such as plates, beads, polymers, etc., is usually functionalized with antibodies that can bind to EVs [32]. Previously, we demonstrated that the polymer nanobody-based method is suitable for purifying EVs from plasma [21]. We have tried to implement the same technology for purifying EVs from urine and compared the “gold standard” method with our developed method. For the optimization of the elution steps, we tried two different approaches (batch and column elution). The batch resulted in more vesicles per mL of isolated sample and different subpopulations, probably due to the longer contact time elution buffer with EVs, as highlighted by the NTA results. Column elution produces a narrower size distribution, potentially enriching for specific vesicle subpopulations, while batch elution captures a broader range of particle sizes with a higher overall yield. These findings suggest that the choice of isolation method can significantly impact the characteristics of the isolated vesicles, which is crucial for downstream applications where vesicle size and purity are critical factors.

At the present and in the absence of molecular data about EV content, we mostly concentrated our analysis on EV phenotypic features. After we saw that batch elution is more effective and accessible and results in a more heterogeneous mixture of uEVs, we further used this approach for immunoaffinity isolation and comparison with the ultracentrifugation method. The relative number of smaller particles in the CFG method is lower than in the IM method, which can be explained by the loss of smaller particles during multiple centrifugation steps specific of this method. Ultracentrifugation is known to potentially cause particle aggregation and contamination with protein aggregates and other urinary components [33]. The choice of EV isolation method may depend on the specific needs of the research. If the aim is to investigate specific subpopulations of EVs or smaller particles, the IM method may be more suitable. The higher protein content and lower protein/lipid ratio in the CFG samples suggest considerable contamination with urinary proteins and aggregates. This can interfere with downstream analyses and reduce the specificity of the isolated EVs. On the other hand, EVs produced by the IM method have a more advantageous protein/lipid ratio, suggesting a sample of higher purity. This higher purity is beneficial for accurate and reliable downstream applications, such as proteomic and lipidomic analyses. This method minimizes contamination and preserves the integrity of the EVs, making it a promising approach for detailed and specific studies. The flow cytometry analysis of uEVs isolated by both ultracentrifugation and immunoaffinity methods reveals distinct surface marker profiles, specifically for CD9, CD63, and CD81. Notably, the new immunoaffinity method successfully isolated vesicles, the quality of which was confirmed by SEM and AFM analyses that showed that isolated vesicles are mainly intact and their diameters are in the appropriate range.

Many kidney and urinary tract diseases are currently diagnosed using non-specific and insensitive biomarkers. For example, serum creatinine, a late and nonspecific marker of kidney dysfunction, is still used to measure changes in kidney function [34]. After years of intensive research, few biomarkers have been approved for clinical use [35,36]. Urinary EVs have excellent potential as a source of multiplex biomarkers. They are readily available in large quantities, non-invasive, and open to frequent longitudinal sampling. EV-based biomarkers in urine are currently investigated for an array of malignancies and other diseases, such as cystinuria, polycystic kidney disease, diabetes, glomerulonephritis, hypertension, or lupus nephritis [12,37,38,39] However, many of the identified candidate biomarkers have not yet been validated in large independent cohorts or tested in more than one laboratory, and the main problem is their purification up to a very pure sample for different specific downstream analyses. The IM protocol we have developed offers an easy-to-perform, fast, and inexpensive method. It results in good yield recovery of vesicles with intact shape and less protein contamination than standard methods. The suitable EV yields necessary for acquiring reliable clinical data still need to be determined, including if the present setting matches such requirements. In contrast, what is already feasible given the robustness of the established purification method is designing downstream analysis with the aim of characterizing the content of purified EVs. Extensive “omisc” on a wider cohort of samples is needed to confirm the utility of the approach, but also to understand how tailoring it to select functionally independent subgroups of EVs. Their availability would simplify the stratification of organ- or disease-specific EVs. Now, the purification platform exploited in this work is extremely flexible, since it can use any nanobody. At the present, the limiting factors are the availability of selective surface biomarkers and of the corresponding specific nanobodies. Blind panning performed with pre-immune libraries has demonstrated to be effective to isolate nanobodies able to discriminate between cells with different proteomic profiles [40] and we expect that this approach, applied to EVs, will allow the recovery of a set of useful reagents. However, exclusive biomarkers are rare and we anticipate that, probably, sorting based on the co-presence of different biomarker configurations will be necessary to separate EV subgroups.

## 4. Materials and Methods

### 4.1. Chemicals

Anti-CD-9 Phytoerythrin (PE)-labelled (clone MM2/57), Alexa Fluor^®^ 488 anti-human CD63 (clone H5C6), and PE/Dazzle™ 594 anti-human CD81 (TAPA-1) antibody (clone 5A6) were from Biolegend (San Diego, CA, USA). Protein Quantification Kit was from Thermo Scientific (Rockford, IL, USA). All other chemicals were of analytical grade and were used without further purification.

### 4.2. Preparation of Microporous Copolymer

The copolymer was prepared according to the procedure previously described, with some modifications [21,23,40,41,42,43]. Briefly, a continuous phase (113 mL of 1% (*w*/*v*) PVP in demineralized water) was heated to 70 °C in a 250 mL reactor equipped with an anchor stirrer. A monomer phase (24.3 g of both the monomer GMA and the cross-linking agent EGDMA (GMA/EGDMA = 2/3), an initiator (0.25 g of AIBN), and an inert phase (32.0 g, 1-tetradecanol/cyclohexanol = 4/1) was added to the continuous phase under stirring at 200 rpm. Five hours later, the reaction was stopped. The obtained copolymer was dried at room temperature (RT) and rinsed five times with ethanol. The particle size distribution was ascertained through the sieve analysis, and copolymer beads ranging in diameter from 125 to 250 µm were employed for the subsequent analysis. Ten grams of diblock copolymer beads were suspended in 250 milliliters of toluene. After adding a ten-fold excess of ethylenediamine in relation to epoxy groups, the reaction was stirred continuously at 25 °C over the course of the entire night. Subsequently, the reaction mixture was heated to 80 °C for six hours and stirred overnight at 25 °C. The polymer particles were isolated, washed with ethanol, and then with water until the pH of the filtrate reached 6.0. The samples were oven-dried at 50 °C for two hours. Elemental analysis was used to determine the concentration of amino groups.

### 4.3. Production of VHHe-GFP

To maximize the capture capacity towards EVs, we used a pool of five heavy chain-only antibodies (VHHs) purified from a naïve pre-immune library obtained by panning on EVs isolated from the supernatant of the cell culture medium [19]. H1 and H6 bind to CD9 antigen, while the other three (G2, D5, and B1) bind to other, not yet determined antigens. VHHs were expressed as fusion proteins with C-terminal eGFP and 6× His tag using a modified pET-14b expression vector [44,45,46] Vectors were transformed in the *E. coli* BL21 (DE3) cells with a plasmid for sulfhydryl oxidase and DsbC. VHHs were purified as previously described, with some minor modifications [47,48]. Briefly, 2 mL of preculture was transferred in 400 mL LB with appropriate antibiotics (100 µg/mL ampicillin, 25 µg/mL chloramphenicol). Bacteria were grown in a shaking incubator at 210 rpm at 37 °C until they reached approximately 0.4 (OD600 nm). The D-arabinose 0.5% (*w*/*v*) was added to induce the expression of DsbC and sulfhydryl oxidase; the temperature was decreased to 30 °C, and bacteria cultures were shaken for 30 min, 210 rpm. Finally, 1 mM Isopropyl β-D-1-thiogalactopyranoside (IPTG) was added in culture to induce the expression of proteins. Bactria cultures were left for overnight expression (18 h) at 21 °C, shaking at 210 rpm. Cell cultures were centrifuged at 4000 rpm, 20 min, and 4 °C the next day. The pellet was resuspended in 20 mL TBS buffer (50 mM Tris, 500 mM NaCl, pH 7.4) after adding lysozyme (0.5 mg/mL final concentration) and DNase (1 U final). Finally, the cell lysate was centrifuged at 13,500 rpm for 20 min at 4 °C. The cell lysate supernatant was loaded on Immobilized Metal Chelate Affinity Chromatography (IMAC) chromatography. Matrix was rinsed with buffer A (50 mM Tris, 150 mM NaCl, 15 mM imidazole, pH 7.4). Unbound proteins were washed with the same buffer A while bound protein was eluted in two steps (first with 50 mM Tris, 500 mM NaCl, 100 mM imidazole and second with 50 mM Tris, 500 mM NaCl, 300 mM imidazole). 

### 4.4. Immobilization of VHHe-GFP on Polymer

Polymethacrylate polymer was washed three times with 0.1 M sodium phosphate buffer, pH 8. Glutaraldehyde (2.5%) was added to the polymer and then incubated for 2 h on RT with occasional stirring. An excess amount of glutaraldehyde was removed from the polymer suspension by rinsing with 0.1 M sodium phosphate buffer, pH 7.0 [42,49]. For VHHs, 100 µg of each of the five constructs were added to the wet polymer, which resulted in a total amount of 500 µg of protein per 1 mL polymer. Immobilization was performed for 24–48 h at 4 °C. The efficiency of immobilization was estimated by measuring the protein concentration before and after immobilization. After, the polymer was rinsed with 0.1 M sodium phosphate buffer, pH 7.0. Free binding sites were blocked with glycine (200 mM, pH 7.0) for 30 min at RT, shaking at 100 rpm. The functional polymer (functionalized with antibodies) can be stored after this step at 4 °C until further use for a maximum of 4 weeks.

### 4.5. Urine Collection

First, morning urine was collected from healthy volunteers (male and female) in a sterile tube and centrifuged twice to remove cells and cell debris, respectively, at 200× *g* for 5 min and then at 2000× *g* for 15 min.

To compare the two methodologies, we collected and pulled four healthy volunteers’ first-morning urine, which resulted in a total of 350 mL. The urine was centrifuged twice at 200× *g* for 5 min and then at 2000× *g* for 15 min to remove cells and cell debris. Then, it was split into two parts: one part for immunoaffinity isolation of extracellular vesicles and the other for ultracentrifugation methods. After these steps, urine can be kept for the short term at 4 °C and for a long time at −80 °C for further analysis.

### 4.6. Optimization of the uEVs Purification Protocol with Nanobodies-Based Chromatography

The functional polymer was first blocked with 5% (*w*/*v*) skimmed milk in PBS to secure all other active sites for 30 min at RT, with shaking. It was washed three times with PBS and further used for purification uEVs. The 35 mL of urine was first filtered on an ultrafiltration cell on a GE Healthcare filter unit (100 kDa) to reduce the sample volume (up to 1 mL). Urine was mixed with polymer and incubated for 1 h with low shaking at RT. After washing with PBS, elution uEVs were performed using the two approaches.

Approach A: Column Elution in 500 µL Fractions

In this approach, the uEVs are eluted from the polymer by adding 200 mM glycine, pH 2.2, as the elution buffer. The elution is performed in 500 µL fractions. Each fraction is collected into a pre-filled tube with an appropriate volume of 1 M Tris, pH 9.1, to neutralize the elution buffer immediately upon collection. The fractions were collected until a negative reaction for proteins on the Bradford spot test.

Approach B: Two-Step Batch Elution

Initially, 400 µL of 200 mM glycine, pH 2.2, is added to the polymer and incubated for 15 min at RT with low shaking. The eluate is then collected into a pre-filled polypropylene (PP) tube with 1 M Tris, pH 9.1, to neutralize the elution buffer. Following the first elution, another 400 µL of the elution buffer is added to the polymer and incubated for an additional 30 min under the same conditions. The eluate from this second step is also collected into the same PP tube pre-filled with 1 M Tris, pH 9.1.

### 4.7. Comparing the “Gold Standard” Method with Optimized Immunoaffinity Methods

From the pulled urine sample, we compared two different isolation processes. Immunoaffinity approach: half of the urine volume was filtered through Amicon Ultra 100 kDa to concentrate and decrease a volume up to 5 mL. Then, 5 mL was split for 1 mL for five individual isolations on polymers with nanobodies and purified according to the uEV purification protocol previously described in part urine collection and optimization of the uEV purification protocol. Ultracentrifugation approach: half of the urine volume, 175 mL, was also filtered through Amicon Ultra 100 kDa. The volume was reduced to 67.5 mL and split into five equal parts of 13.5 mL due to the maximum volume of the ultracentrifuge tube. The urine was centrifugated at 20,000× *g* to remove potential protein aggregates and then at 100,000× *g* to pellet the urine EVs. The EVs were resuspended in PBS and then applied in downstream analysis. In the isolates, proteins, lipids, particle number and size, and marker presence were determined.

### 4.8. uEVs Quantification and Morphology

The size distribution and quantity of uEVs were assessed using Nanoparticle Tracking Analyzer ZetaView Quatt PMX-430 (ParticleMetrix, Inning am Ammersee, Germany) with ZetaView software version 8.05.16 SP3 (NTA). The instrument was set (camera/laser alignment and focus optimization) using 100 nm polystyrene beads according to the manufacturer’s instructions. Samples were diluted in PBS to reach optimal particle count per frame. A blue laser (488 nm) was used for measurements in scatter mode. Camera settings were: sensitivity of 78, shutter speed of 100, and a frame rate of 30 frames/s. Post-acquisition parameters were: minimal area of 10, maximal area of 1000, and minimum brightness of 30.

The obtained raw data (the number of detected particles for each EV size) are plotted using Excel (Microsoft Office 16).

### 4.9. Morphology

#### 4.9.1. Scanning Electron Microscopy

We used scanning electron microscopy (SEM) to determine the morphology of purified EVs. uEVs were loaded on metal stubs and dried with air. Before starting SEM analysis, samples were coated with a gold layer using a sputter coater (Polaron SC503, Fisons Instruments, Glasgow, Scotland). Tescan Fe-SEM Mira 3XMU (Tescan, Brno, Czech Republic) characterized the uEVs’ morphology.

#### 4.9.2. Atomic Force Microscopy

The surface morphology was investigated by atomic force microscopy (AFM) with a NanoScope 3D (Veeco, Plainview, NY, USA) microscope operated in taping mode under ambient conditions. Etched silicon probes with a spring constant of 20–80 Nm^−1^ were used. Image analysis was performed using Nanoscope image processing software (v1.40r1). Before the morphological tests, the mica substrate was mechanically polished with adhesive tape. Ten μL of the prepared suspension EVs was applied to a polished mica substrate and air-dried.

### 4.10. Determining Surface Marker of uEVs

#### Flow Cytometry

Samples were prepared for flow cytometry analysis as previously described with slight modifications. Briefly, 30 µL of 1% suspension of beads was mixed with 4–40 µg vesicle proteins and incubated at 4 °C overnight with shaking. The next day, beads were blocked with 200 mM glycine for 30 min and then with 5% skimmed milk in PBS before being washed with PBS to remove all retained blocking solutions. In the end, the commercial anti-CD-9, anti-CD-81, and anti-CD-63 antibodies were added to three independent portions of the initial bead batch, each diluted 1:5 and incubated for 1 h. The resulting three samples were analyzed separately by BD FACSCalibur (BD Biosciences, Franklin Lakes, NJ, USA). For excitation, a blue state laser (488 nm) was used. Emissions were followed in the next wavelengths: anti-CD-61 (AlexaFluor 488, FL1, 525 nm), anti-CD-9 (PE, FL2, 561 nm), anti-CD-81 (PE/Dazzle, FL3, 620 nm). The positive events were measured against non-coated beads blocked with milk. As a negative control, Triton X-100 (1% solution) was added to all samples in the ratio 1:1 (final concentration 0.5%) and incubated for one hour at RT, before the measurements were repeated as previously described.

### 4.11. Determination of uEVs Protein and Lipid Content

The amount of EV proteins was evaluated with a Micro BCA Protein Assay Kit (Thermo Scientific^TM^) using a BSA as a standard.

The colorimetric sulfophosphovanilin assay (SPV) was used to determine lipid content in uEVs using the procedure previously described [21,50].

### 4.12. Statistical Analysis

For data analysis, we used GraphPad Prism v.9. We used the Wilcoxon matched-pairs signed rank test to compare different parameters of EV subpopulations. *p* values of less than 0.05 were considered statistically significant.

## 5. Conclusions

In this work, we have successfully used nanoantibodies displayed on polymethacrylate carriers to purify EVs from urine. Compared to the “gold standard” method, we have demonstrated in this paper that the immunoaffinity method can be used to successfully purify EVs that have specific morphological features. Using NTA, the number of vesicles was determined and the yields were comparable to those obtained using the gold standard protocol. The process is fast, scalable, reproducible, cost-effective, easy to perform, and produces reproducible results, whereas the risk of product contamination with proteins and other components usually detected in samples purified with standard methods is significantly lower.

## Figures and Tables

**Table 1:** We measured the average size and concentration of vesicles using NTA.

	Volume of Urine (mL)	Yield (Total Number EV)	Average Size (nm)
Isolate 1 (Column elution)	35	8.72 × 10^10^	100.12 ± 15.9
Isolate 2 (Batch elution)	35	4.06 × 10^11^	124.8 ± 2.47

**Table 2:** Comparison of protein and lipid content, and protein/lipid ratios of samples obtained using two different isolation methods: ultracentrifugation isolation of extracellular vesicles from urine (CGF) and immunoaffinity isolation of extracellular vesicles from urine (IM). Values with different letters are significantly different.

Type of Isolation Methods	Proteins (mg/mL)	Lipids (mg/mL)	Proteins/Lipids
CFG	1.349 ± 0.180 ^a^	0.522 ± 0.077 ^c^	2.659 ± 0.716
IM	0.279 ± 0.035 ^b^	0.074 ± 0.011 ^d^	3.819 ± 0.531

**Table 3:** NTA analysis of particles after purification of urinary EVs with ultracentrifugation (CFG) and the immunoaffinity method (IM).

Type of Sample	Yield (Total Number EV)	Average Size (nm)
Urinary EVs by CFG	2.8 × 10^11^	140.4
Urinary EVs by IM	1.47 × 10^11^	127.45

## Data Availability

Data is contained within the article and Supplementary Materials.

## Supplementary Materials

The following supporting information can be downloaded at: https://www.mdpi.com/article/10.3390/ijms25158004/s1.

## Abbreviations

EVs—extracellular vesicles; AKI—acute kidney injury; uEVs—urinary extracellular vesicles; SEC—size exclusion chromatography; CFG—ultracentrifugation method; IM—immunoaffinity method; GFP—green fluorescence protein; VHH—single-domain antibodies; SEM—scanning electron microscopy; AFM—atomic force microscopy; NTA—nanoparticle tracking analysis.
