# Supplementary material for: Urinary Extracellular Vesicles as a Readily Available Biomarker Source: A Simplified Stratification Method
Source: Int J Mol Sci. 2024 Jul 23;25(15):8004. doi: 10.3390/ijms25158004 (PMC11311997; doi:10.3390/ijms25158004)
Supplement: Supplementary file 1 [file ijms-25-08004-s001.zip › ijms-3099892-supplementary.pdf]

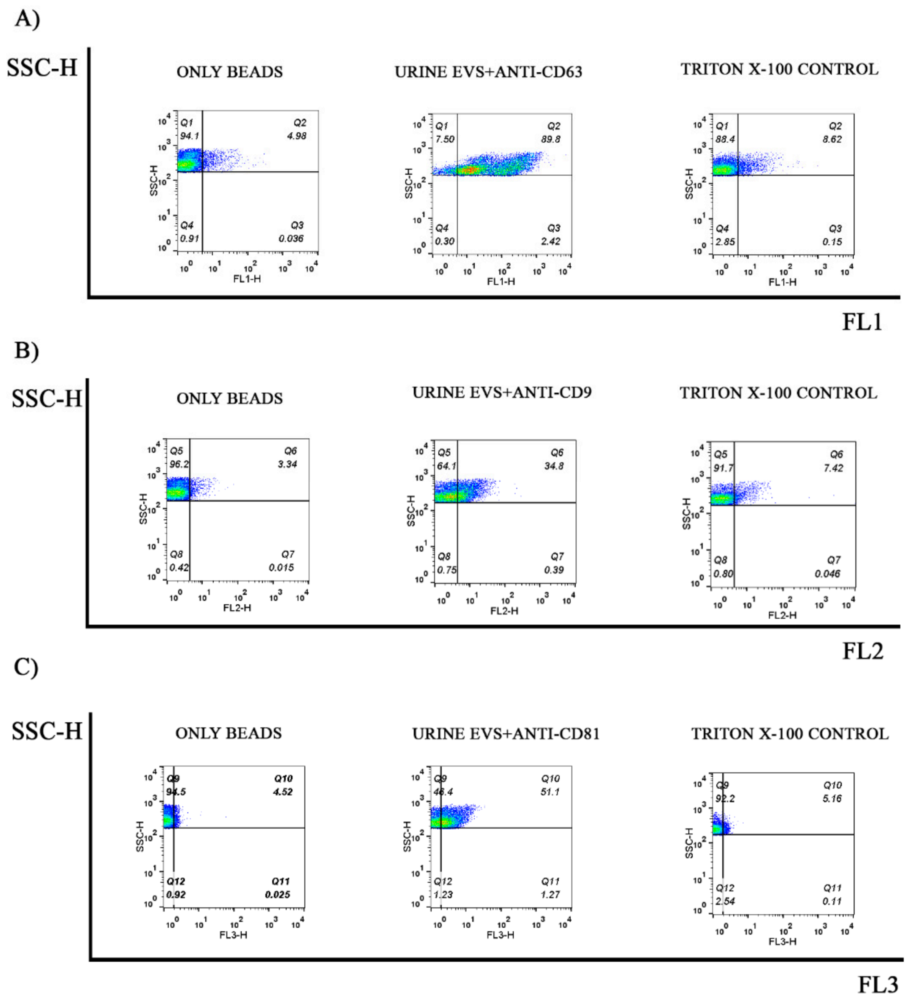

Suppl. Figure S1. Flow cytometry analysis of uEVs using commercial antibodies against surface markers.

A) Staining using anti-CD63 antibodies; B) Staining using anti-CD9 antibodies; C) Staining using anti-CD81 antibodies. Triton X-100 treatment was used as a negative control, Gates were set according to the autofluorescence of uncoated beads.
